# Calcium‐based biomineralisation remodels the pathogen‐host interface

**DOI:** 10.1002/ctm2.70736

**Published:** 2026-07-22

**Authors:** Linglin Fu, Yilin Wang, Wenting Zhang, Yuqi Jin, Wanying Zhang, Feng Xu

**Affiliations:** ^1^ Department of Infectious Diseases The Second Affiliated Hospital Zhejiang University School of Medicine Hangzhou China; ^2^ Institute of Translational Medicine Zhejiang University Hangzhou China; ^3^ Institute of Fundamental and Transdisciplinary Research Zhejiang University Cancer Centre Zhejiang University Hangzhou China; ^4^ The Centre for Translational Research The Second Affiliated Hospital and Institute of Translational Medicine Zhejiang University Hangzhou China

1

Biomineralisation refers to the natural process through which organisms regulate the formation and deposition of inorganic minerals.^1^ Inspired by this phenomenon, engineered biomineralisation has emerged as a materials strategy for constructing mineral coatings on cellular and microbial surfaces, thereby regulating their stability, resistance to environmental stress, delivery efficiency, and immune interactions.^2^ Based on mineral composition, surface mineralisation can be broadly classified into calcium‐based mineralisation (e.g. calcium phosphate and calcium carbonate), manganese‐based mineralisation (e.g. MnO_2_), iron‐based mineralisation (e.g. Fe_3_O_4_ and FeS) and silicification (silica deposition), each with distinct chemical properties and biological effects. Among these, calcium‐based mineralisation is distinguished by its favourable accessibility and biosafety in vivo. Calcium is one of the most abundant mineral elements in the human body, where physiological systems governing calcium phosphate deposition and remodelling are already in place.^3^ Calcium phosphate and calcium carbonate constitute key components of bones and teeth, and their degradation products can be readily absorbed and metabolically utilised in vivo. Compared with other mineralisation systems, calcium‐based mineralisation is better suited for in situ formation in vivo and holds greater promise for biocompatible and clinically translatable applications.

In recent years, calcium‐mediated therapeutic strategies have gained considerable interest in oncology. Localised induction of calcium salt deposition in tumour tissues has been reported to disrupt cellular metabolism, damage membrane integrity and promote cell death, thereby suppressing tumour growth.^4^ These studies support the therapeutic feasibility of inducing calcification in vivo. Nevertheless, extending this concept from tumours to bacterial infections is not straightforward. Compared with tumour cells, pathogenic bacteria are smaller, possess structurally complex surfaces and reside in rapidly changing infection microenvironments, making selective bacterial calcification at infectious lesions particularly challenging.

Recently, we advanced this concept by achieving the first targeted in situ calcification of methicillin‐resistant *Staphylococcus aureus* (MRSA) within living hosts.^5^ Using a wall teichoic acid‐recognising antibody to deliver polysialic acid to the bacterial surface, we promoted local calcium enrichment and induced the formation of a calcified coating on MRSA within infectious foci. In contrast to traditional antibiotics targeting specific molecular mechanisms, bacterial surface calcification would impose mechanical restrictions on pathogens by forming a calcium‐carbonate shell around them, limiting the molecular exchange of bacteria with their environment and thus also impacting energy metabolism, membrane transport, virulence regulation, quorum sensing and biofilm formation. This multilevel combination of physical encapsulation and functional suppression may, in principle, diminish the threat of resistance escape through single‐target mutations. Most importantly, bacterial surface calcification does more than limit pathogenicity; it alters recognition by the host immune response. Mechanistically, calcified bacteria induce calcium‐binding proteins with damage‐associated molecular pattern‐like activity, including S100A8/S100A9 (calprotectin), thereby potentiating innate immune responses in the monocyte‐macrophage compartment and enhancing bacterial clearance. Therefore, the significance of calcification‐based therapy lies not merely in creating an antibacterial barrier but in remodelling the pathogen‐host interface, shifting pathogens from an immune‐evasive state toward one that is more susceptible to immune recognition and elimination. Our work represents a critical proof of concept for translating biomineralisation from in vitro engineering platforms to in vivo pathogen remodelling.

Despite this therapeutic promise, in vivo mineralisation still faces several translational barriers, including delivery specificity and efficiency, control over mineral deposition, potential ectopic calcification, and long‐term safety. Moreover, because pathogen surfaces and infection microenvironments vary substantially across species, further efforts are needed to establish calcification platforms that are broadly applicable, robust, and precisely controllable across diverse pathogens.

In addition to the therapy of existing infections, bacterial mineralisation might be an attractive platform for infection prevention via whole‐cell vaccines as well. A perfect whole‐cell bacterial vaccine would attenuate pathogenicity without losing its native antigenic repertoire, a balance that mineralisation could potentially achieve by suppressing bacterial viability while retaining pathogen‐associated surface antigens. Recent works revealed that the use of a vaccine with *S. aureus* mineralised by manganese was found to reduce the toxic effects, as well as promote innate immune responses and antigen presentation.^6^ Likewise, calcium phosphate mineralisation of an attenuated *Streptococcus pneumoniae* vaccine showed improved bacterial stability as well as stronger humoral and Th17 immune responses.^7^ Collectively, these findings suggest that mineralisation can simultaneously reduce bacterial virulence and enhance vaccine immunogenicity. A key question moving forward is whether mineralised bacteria can induce trained immunity.^8^ While continued mineral coating degradation could potentially extend innate immune stimulation, whether this step is enough to induce durable metabolic and epigenetic reprogramming and finally lead to heterologous protection remains to be shown.

Taken together, targeted bacterial calcification brings the materials concept of cell‐surface mineralisation into antibacterial therapy for drug‐resistant infections and demonstrates that pathogens can be selectively mineralised in vivo. The significance of this strategy lies not only in offering an antibiotic‐independent approach to infection control but also in establishing pathogen surfaces as engineerable therapeutic interfaces. With further advances in targeted recognition, controlled mineralisation, and infection immunology, calcium‐based biomineralisation may serve as a bridge between anti‐infective therapy and vaccine development, opening new translational avenues for combating drug‐resistant bacterial infections.

## AUTHOR CONTRIBUTIONS

Linglin Fu and Yilin Wang contributed equally to this work and drafted the manuscript. Wenting Zhang and Yuqi Jin contributed to the review and revision of the manuscript. Wanying Zhang and Feng Xu conceptualized and supervised the work and critically revised the manuscript. All authors reviewed and approved the final version of the manuscript.
